# Redox Metabolism-Associated Molecular Classification of Clear Cell Renal Cell Carcinoma

**DOI:** 10.1155/2022/5831247

**Published:** 2022-01-21

**Authors:** Xiangling Wei, Weiming Deng, Zhanwen Dong, You Luo, Xiao Hu, Jinhua Zhang, Zhenwei Xie, Tong Zheng, Yuqin Tan, Zuofu Tang, Heng Li, Ning Na

**Affiliations:** ^1^Department of Kidney Transplantation, The Third Affiliated Hospital of Sun Yat-sen University, Guangzhou 510630, China; ^2^The First Affiliated Hospital, Department of Urology, Hengyang Medical School, University of South China, Hengyang, Hunan 421001, China

## Abstract

Clear cell renal cell carcinoma (ccRCC) is the most common subtype of renal cell carcinoma. Redox metabolism has been recognized as the hallmark of cancer. But the concrete role of redox-related genes in patient stratification of ccRCC remains unknown. Herein, we aimed to characterize the molecular features of ccRCC based on the redox gene expression profiles from The Cancer Genome Atlas. Differentially expressed redox genes (DERGs) and vital genes in metabolism regulation were identified and analyzed in the ccRCC. Consensus clustering was performed to divide patients into three clusters (C1, C2, and C3) based on 139 redox genes with median FPKM value > 1. We analyzed the correlation of clusters with clinicopathological characteristics, immune infiltration, gene mutation, and response to immunotherapy. Subclass C1 was metabolic active with moderate prognosis and associated with glucose, lipid, and protein metabolism. C2 had intermediate metabolic activity with worse prognosis and correlated with more tumor mutation burden, neoantigen, and aneuploidy, indicating possible drug sensitivities towards immune checkpoint inhibitors. Metabolic exhausted subtype C3 showed high cytolytic activity score, suggesting better prognosis than C1 and C2. Moreover, the qRT-PCR was performed to verify the expression of downregulated DERGs including ALDH6A1, ALDH1L1, GLRX5, ALDH1A3, and GSTM3, and upregulated SHMT1 in ccRCC. Overall, our study provides an insight into the characteristics of molecular classification of ccRCC patients based on redox genes, thereby deepening the understanding of heterogeneity of ccRCC and allowing prediction of prognosis of ccRCC patients.

## 1. Introduction

Kidney cancer is the sixth most commonly diagnosed cancer in the male and the eighth in the female, accounting for 5% and 3% of all cancers in the USA in 2019 [1]. Among renal cell carcinoma (RCC) which accounts for more than 90% of the kidney cancer, clear cell renal cell carcinoma (ccRCC) is the most common subtype and accounts for the majority of death from RCC [2, 3]. Due to the heterogeneity, preference for metastasis, and complex metabolic processes of ccRCC, significant survival benefits have not been achieved in present treatment for these patients [4]. Emerging studies seek to develop a model or classification for prognosis prediction, but it still requires improvement in predictive accuracy or better molecular classification of ccRCC. Metabolism reprogramming is thought as the hallmark of cancer and characterized by dysregulated uptake of glucose and amino acids, increased demand of more nutrients and nitrogen, and use of glycolysis and tricarboxylic acid (TCA) cycle intermediates for NADPH [5]. Redox balance plays a key role in promoting tumorigenesis and metastasis [6]. The redox metabolism signaling starts with the production of reactive oxygen species (ROS) due to incomplete reduction of oxygen molecules for mitochondrial ATP generation or response to growth factor signaling by oxidase such as NADPH oxidase [7]. During tumorigenesis, cancer cell increases metabolic activity and ROS production, which subsequently activates downstream signaling pathways for proliferation and survival [6]. Recently, the metabolism-associated molecular classification for hepatocellular carcinoma and colorectal cancer has been reported as prognostic and promising classifiers [8, 9]. In ccRCC, it has been reported that cancer cell depends on glutathione redox metabolism for removal of ROS [10]. In addition, a redox-related lncRNA signature and a redox-related gene signature have been proposed as efficient prognostic tools for ccRCC. These collectively indicated the vital role of redox metabolism in ccRCC, but no research about redox-associated molecular classification has been conducted in ccRCC.

In this study, we aimed to discover subclasses of ccRCC based on redox genes using RNA sequencing data of 530 patients from The Cancer Genome Atlas (TCGA). We firstly identified and analyzed differentially expressed redox genes (DERGs) and key metabolism-regulated genes in ccRCC compared with adjacent normal tissue. We stratified patients based on redox gene expression profiles by consensus clustering analysis and investigated functional differences among clusters. Moreover, we clarified the association of clusters with immune cell infiltration, immune markers, mutation, and immunotherapy responses. Overall, our study provided a new idea for molecular classification of ccRCC patients.

## 2. Materials and Methods

### 2.1. Data Collection and Processing

Level 3 RNA-seq transcriptome data, corresponding clinical information, and somatic mutation data (MAF files) of 539 ccRCC samples and 72 normal samples were retrieved from TCGA (http://cancergenome.nih.gov/). Raw counts and fragments per kilobase of transcript per million mapped reads (FPKM) were used for differential expression analysis and further bioinformatic analysis, respectively. Patients with incomplete clinical information were excluded, and finally, 530 ccRCC samples and 72 normal samples were used for further research. TCGA Batch Effects Viewer (http://bioinformatics.mdanderson.org/tcgambatch/), allowing identification and quantification of the batch effects presented in a given TCGA dataset, was used to analyze the homogeneity of ccRCC samples. All ccRCC samples used in this study were homogenous.

A total of 174 genes directly and indirectly involved in redox metabolism were obtained from Benfeitas et al. [11]. These genes are mainly responsible for antioxidant and ROS-dependent activities, producing compounds with redox characteristics, folate metabolism, malate metabolism, and transcription factors associated with oxidative stress [11]. Redox genes with median FPKM value > 1 across patients with ccRCC were selected for further analysis.

### 2.2. Identification of Differentially Expressed Redox Genes

To identify differentially expressed genes (DEGs), raw counts of ccRCC samples and normal samples were analyzed using the ‘edgeR' package of R software [12]. Additionally, to identify cluster-specific genes, we performed differential expression analyses using the ‘edgeR' package in ccRCC samples between one cluster and remaining clusters. Genes with ∣ log (fold change) | ≥1 and FDR adjusted *P* < 0.05 (Benjamini & Hochberg method) were considered DEGs.

DERGs were common genes in DEGs and redox genes, and their prognostic value in ccRCC were analyzed. Additionally, we obtained a list of key genes in regulating metabolism [13, 14] and then analyzed their expression as well as prognostic value in ccRCC. The GEPIA2 portal (http://gepia2.cancer-pku.cn/#index), which was a database integrating RNA-seq expression data and clinical data from TCGA, was used to perform survival analysis of selected genes [15]. In the survival analysis, all ccRCC patients were divided into two groups according to the cutoff determined by 50% of the gene expression. The survival analyses were performed using the Kaplan-Meier method and log-rank test. Log-rank *P* < 0.05 was used as the significance cutoff. For multiple comparison in the survival analysis, adjusted *P* < 0.05 (Benjamini & Hochberg method) was used as the significance cutoff. The hazard ratio was calculated for each analysis. Additionally, the Human Protein Atlas (HPA) database (https://www.proteinatlas.org/) was used to analyze the protein expression of selected proteins in ccRCC tissue compared with normal kidney tissue.

### 2.3. Hierarchical Clustering Analysis

In order to identify ccRCC clusters with similar molecular function, tumor samples were divided into different clusters by using the ‘ConsensusClusterPlus' R package based on the normalized expression profile of 139 redox genes [16]. The resampling method was used to sample 80% of the patients for 50 times, and the Euclidean distance was used to estimate similarity among samples. All ccRCC samples were clustered into *k* (2–6) groups. We determined the optimal number of clusters according to clinical consideration and cumulative distribution function (CDF). Then, we distributed all ccRCC patients into different clusters and investigated their clinical traits. The principal component analysis (PCA) was performed to analyze the expression differences among clusters. Cluster-specific genes were defined as DEGs only in one cluster.

### 2.4. Functional Enrichment Analysis

To determine the functional differences among clusters, the gene set variation analysis (GSVA) in terms of 113 metabolism-associated signatures which were obtained from Rosario et al. [17], was performed using the ‘GSVA' R package [18]. The differences between samples were analyzed by using the ‘Limma' R package with a cutoff of ∣ log (fold change) | ≥0.2 and adjusted *P* < 0.05 [19]. In addition, to explore the potential function and signaling pathways of cluster-specific genes, the gene ontology (GO) and Kyoto Encyclopedia of Genes and Genomes (KEGG) pathway analyses were performed using ‘ClusterProfiler' R package [20]. Terms with adjusted *P* < 0.05 were considered statistically significant.

### 2.5. Estimation of the Immune Infiltration

The CIBERSORTx algorithm [21], which decoded cellular heterogeneity and estimated the abundance of 22 immune cell types in complex tissue, was used to estimate the cell abundance in each sample using the RNA-seq data of KIRC from TCGA. Significant results (*P* < 0.05) were selected for subsequent analysis. The abundances of immune cells were compared among clusters.

### 2.6. Association of Clusters with Mutation, TMB, CYT, and Neoantigen

Genetic somatic mutation data of KIRC from TCGA were used to analyze the differences among clusters. The ‘maftools' R package was used to analyze differences of genetic mutation among clusters [22]. The tumor mutation burden (TMB) was calculated from somatic mutation frequency by using data from the ‘TCGAmutations' R package [23]. Neoantigen and aneuploidy scores of KIRC samples were retrieved from previous study [22, 24]. The cytolytic activity (CYT) score represented the activity of cytotoxic T cells, and it was estimated by the mean mRNA expression of granzyme A (GZMA) and perforin 1 (PRF1) [24].

### 2.7. Evaluation of the Benefit of Three Clusters from Immunotherapy

The data of the immunotherapy efficacy from melanoma patients were used to predict the immune checkpoint inhibitors' efficacy of our clusters [25]. Melanoma patients were treated with cytotoxic T-lymphocyte-associated protein-4 (CTLA-4) blockade and programmed cell death protein-1 (PD-1) blockade. SubMap (subclass mapping method) is an unsupervised method to estimate the significance of the association between subclasses in two independent datasets which were comprised of multiple tissue types or datasets from various platforms [26]. SubMap analysis in GenePattern (https://cloud.genepattern.org/gp/pages/login.jsf) was performed to evaluate the potential of our clusters' responses to immunotherapy by comparing the similarity of gene expression profiles between our subclasses and melanoma patients.

### 2.8. Quantitative Real-Time PCR (qRT-PCR)

Total RNA was isolated from the ccRCC cell line A498 and normal human renal tubule epithelial cell line HK-2 using TRIzol reagent (Invitrogen, Thermo Fisher Scientific, United States). The RNA was then used to perform reverse transcription using the 1st Strand cDNA Synthesis (+gDNA wiper) Kit (Vazyme, Nanjing, China). The ABI QuantStudio™ 5 (Thermo Fisher Scientific, Inc.) and SYBR Green PCR Master Mix (Vazyme, Nanjing, China) were used in qRT-PCR based on the manufacturer's guideline. The relative mRNA expression levels were calculated using the 2-*ΔΔ*Ct method with normalization to GAPDH mRNA. The primers used in this study are listed in Supplementary Table S1.

### 2.9. Statistical Analysis

Statistical analyses were performed using GraphPad Prism software version 8 (GraphPad Software, San Diego, CA, USA), SPSS 25 (SPSS, Inc., Chicago, IL, USA), and R software 3.5.2. Survival analysis and categorical variables among clusters were compared by the chi-square test and Fisher's exact test. Differences between two groups were compared using Student's *t*-test or the Mann–Whitney test. The qRT-PCR results were presented as mean ± standard deviation. All the tests were two-sided, and a value of *P* < 0.05 was considered statistically significant. Benjamini & Hochberg method was used to adjust *P* values.

## 3. Results

### 3.1. Identification of DERGs in the ccRCC

**Figure 1:**
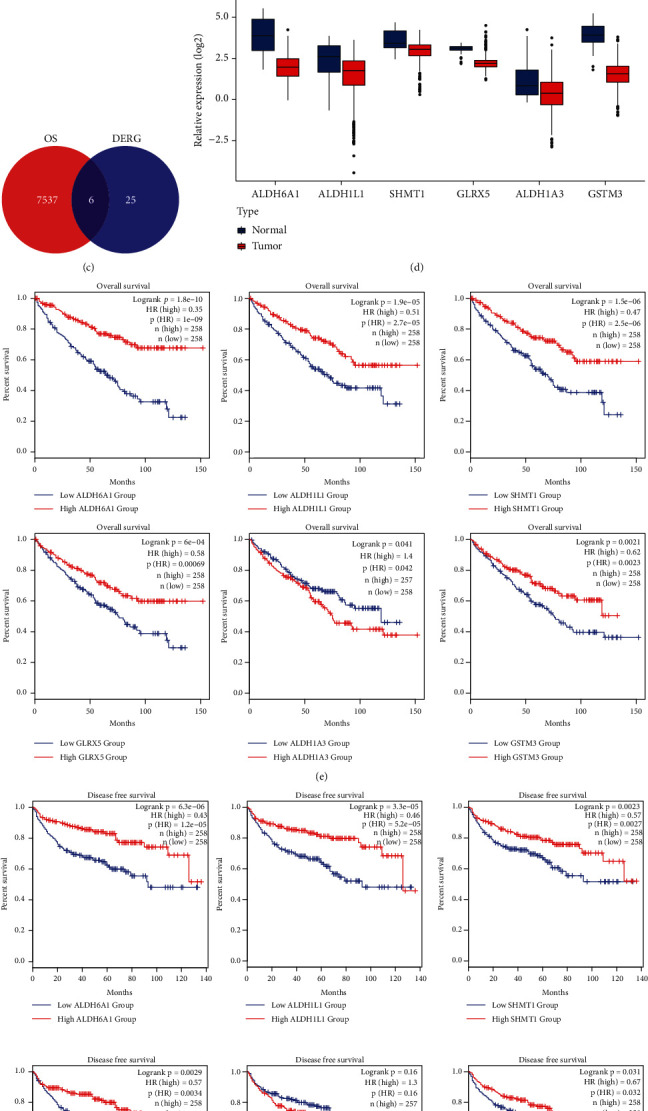
Identification of differentially expressed redox genes (DERG) in the ccRCC. (a) Venn diagram showing 31 DERGs in 2,684 differentially expressed genes and 139 redox genes. (b) The heat map displaying the expression profile of 31 DERGs in ccRCC tissue (T) and normal tissue (N) in TCGA database. (c) Venn diagram showing six prognostic DERGs among prognostic genes in ccRCC and DERGs. (d) Expression levels of six prognostic DERGs in ccRCC compared with normal tissues. Expression data were normalized by log2 transformation. ^∗∗∗^*P* < 0.001. (e, f) Overall survival and disease-free survival analyses of patients with six prognostic DERGs in TCGA database using the GEPIA2 portal. The Kaplan-Meier method and log-rank test were used in the survival analysis.

To analyze the difference of gene expression between the ccRCC and normal tissue, we firstly identified 2,684 DEGs with ∣log (fold change)  | ≥1 and FDR adjusted *P* < 0.05 in ccRCC compared with normal tissue using TCGA data. A list of 174 redox genes were obtained from Benfeitas et al. [11], and 139 redox genes with median FPKM value > 1 were selected for subsequent analysis (Supplementary Table S2). A total of 31 DERGs among DEGs were identified (Figure 1(a)), and their mRNA expression levels were shown in the heatmap (Figure 1(b); Supplementary Table S3). To identify prognostic DERGs, overall survival (OS) analysis was performed and 7,543 prognostic genes were found in ccRCC. Then, six prognostic DERGs including ALDH6A1, ALDH1L1, SHMT1, GLRX5, ALDH1A3, and GSTM3 were identified (Figure 1(c)), and all genes were significantly downregulated in ccRCC patients (*P* < 0.001; Figure 1(d)). Survival analysis indicated that reduced expression of these genes except ALDH1A3 significantly correlated with poor prognosis of both OS and disease-free survival (DFS) in ccRCC patients (log-rank *P* < 0.05; Figures 1(e) and 1(f)).

**Figure 2:**
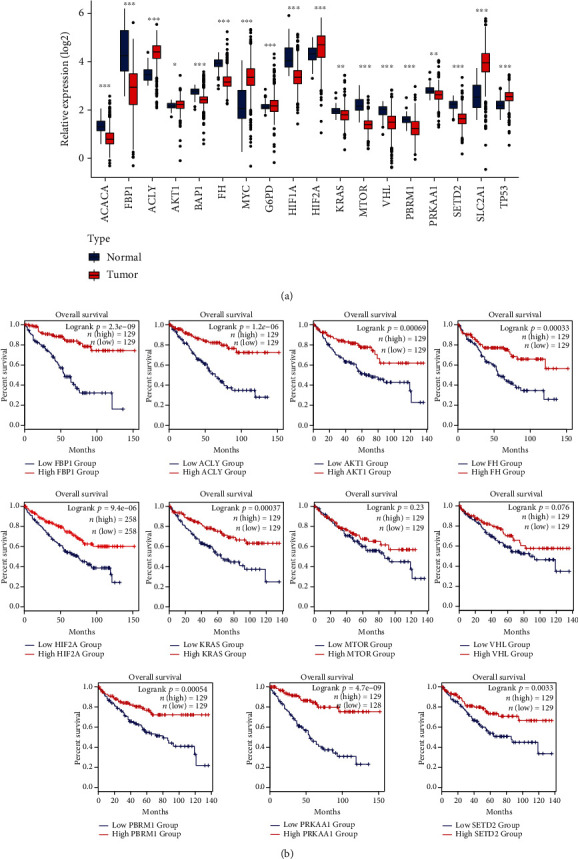
The expression profile and survival analysis of vital genes in metabolism regulation. (a) The expression of vital genes in ccRCC compared with normal tissues using TCGA data. Expression data were normalized by log2 transformation. ^∗^*P* < 0.05, ^∗∗^*P* < 0.01, and ^∗∗∗^*P* < 0.001. (b) Overall survival analyses of vital genes using the GEPIA2 portal. The Kaplan-Meier method and log-rank test were used in the survival analysis.

Moreover, we obtained vital genes for metabolism regulation from Tong et al. and Massari et al. (Supplementary Table S4) [13, 14] and analyzed their expression levels and prognostic value in ccRCC. Specifically, ACACA, FBP1, BAP1, FH, HIF1A, KRAS, MTOR, VHL, PBRM1, PRKAA1, and SETD2 were downregulated, while ACLY, AKT1, MYC, G6PD, HIF2A, SLC2A1, and TP53 were upregulated in ccRCC tissues compared with normal tissue (Figure 2(a)). Survival analysis showed that decreased expression of FBP1, ACLY, AKT1, FH, HIF2A, KRAS, MTOR, VHL, PBRM1, PRKAA1, and SETD2 correlated with worse OS (log-rank *P* < 0.05; Figure 2(b)). Thus, aberrantly expressed and prognostic genes in redox metabolism and metabolism regulation had been identified and suggested possible function in ccRCC.

### 3.2. Identification of Three Clusters in ccRCC Based on Redox Genes

**Figure 3:**
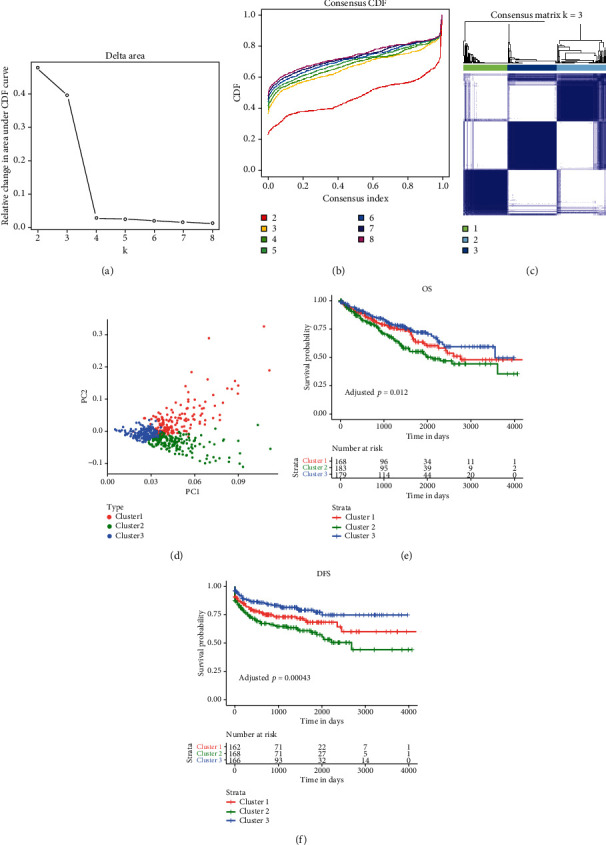
Classification of patients into three clusters by consensus clustering analysis. Consensus clustering CDF (a) and relative change in area under CDF curve (b) for *k* = 2-8. (c) Clustering matrix when *k* = 3. (d) Principal component analysis of gene expression of three clusters. (e, f) Survival analysis using the Kaplan-Meier method for three clusters of ccRCC. The adjusted *P* value (Benjamini & Hochberg method) was calculated with the log-rank test by comparing three clusters.

Based on the expression similarity of 139 redox genes in TCGA, the consensus clustering for combining *K*-means clustering of ccRCC samples was performed. Combining clinical consideration and CDF, *k* = 3 was selected as the optimal value with clustering increasing from *k* = 2 to *k* = 8 (Figures 3(a) and 3(b)). The consensus matrix heat map kept sharp and distinct boundaries when *k* = 3, suggesting robust clustering for all samples (Figure 3(c)). Patients with ccRCC were divided into three clusters (three clusters were assigned C1, C2, and C3), and the PCA showed distinct differences among clusters (Figure 3(d)). Survival analysis of OS and DFS among clusters showed relatively evident distinction (Figures 3(e) and 3(f)). C2 had shorter OS (adjusted *P* < 0.01) and DFS (adjusted *P* < 0.001) than C3, while no significant difference was seen between C1 and C3 in both OS or DFS. Overall, ccRCC patients could be classified into three clusters with significant differences in gene expression and survival based on the redox gene expression profile.

### 3.3. Functional Annotation of Clusters and Cluster-Specific Genes

**Figure 4:**
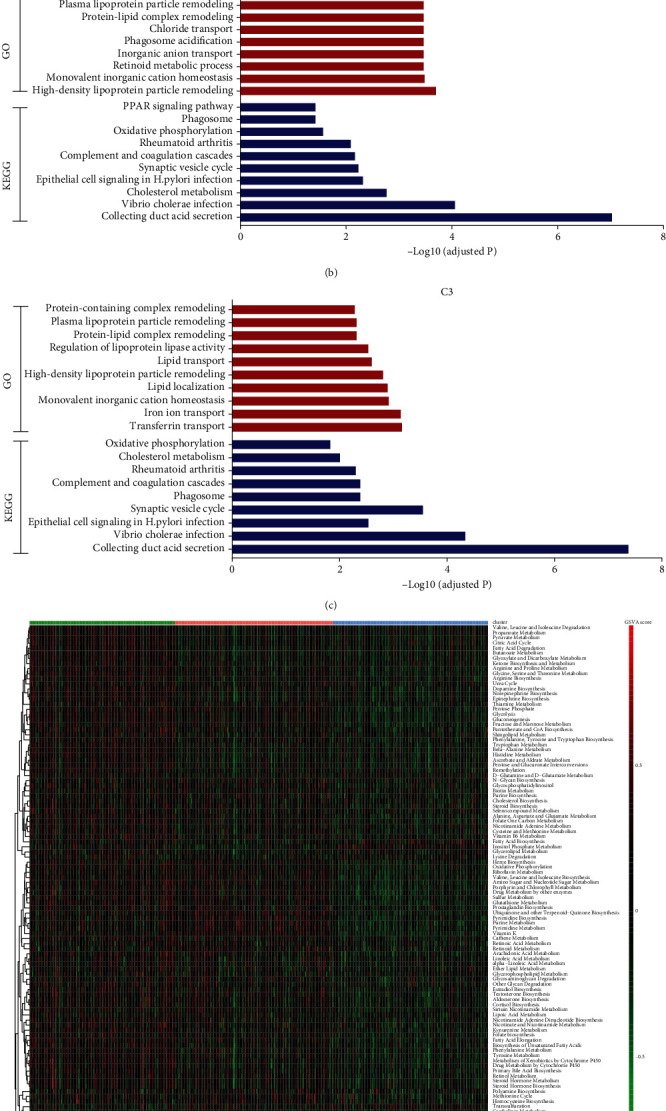
Functional annotation of three clusters and identification of cluster-specific genes. (a–c) GO and KEGG analyses of C1, C2, and C3. (d) The heat map showing GSVA score (pathway enrichment score) of significant metabolic signatures among three clusters (terms with adjusted *P* < 0.05). The color bar showing GSVA scores of metabolic signatures in each sample. Metabolic signatures with adjusted *P* < 0.05 were shown. (e) Venn diagram showing 8, 22, and 23 cluster-specific genes for C1, C2, and C3, respectively. Cluster-specific genes were defined as the DEGs only in one cluster.

To better characterize three ccRCC clusters, we sought to identify cluster-specific genes and analyzed involved function and signaling pathways of each cluster. Differential expression analyses were performed for each cluster compared with remaining clusters. There were 32 DEGs for C1 compared with C2 and C3, 74 DEGs for C2 compared with C1 and C3, and 72 DEGs for C3 compared with C1 and C2. Then, these genes were selected for functional enrichment analyses to clarify the function of each cluster. The results showed that C1 mainly enriched in lipid metabolism including cholesterol metabolism, fat digestion, glycolipid and cellular lipid catabolic processes, triglyceride metabolic process, and PPAR signaling pathway (Figure 4(a)). The potential function of C2 and C3 was similar but C3 mainly enriched in transferrin transport and iron ion transport (Figure 4(b) and 4(c)). To further identify the metabolic differences among clusters, GSVA analysis in terms of metabolic pathways obtained from Rosario et al. [17], showed evident distinction in three clusters. Each sample got a GSVA score (pathway enrichment score) for each metabolic pathway. Significantly differential metabolic pathways (adjusted *P* < 0.05) were identified by performing differential analysis among clusters (Figure 4(d)). Pathways with the highest GSVA score were defined as cluster-specific metabolic pathways. C1 and C2 had 81 and 23 cluster-specific metabolic pathways while C3 had only 2 cluster-specific metabolic pathways. Therefore, we regarded C1 and C3 as metabolic active and metabolic exhausted subtype, respectively. And C2 was seen as the intermediate subtype. As shown in Supplementary Table S5, C1 had the most differential metabolic signatures including glucose, lipid, and protein metabolism. On the other hand, C2 was associated with purine metabolism, pyrimidine metabolism, and glycan synthesis. Moreover, to identify cluster-specific genes which were considered differentially expressed genes in only one cluster, we identified 8 cluster-specific genes for C1, 22 for C2, and 23 for C3 after exclusion of common genes (Figure 4(e); Supplementary Table S6). In summary, these results revealed potential function and involved pathways of three clusters.

### 3.4. Clinicopathological Characteristics of the ccRCC Clusters

**Figure 5:**
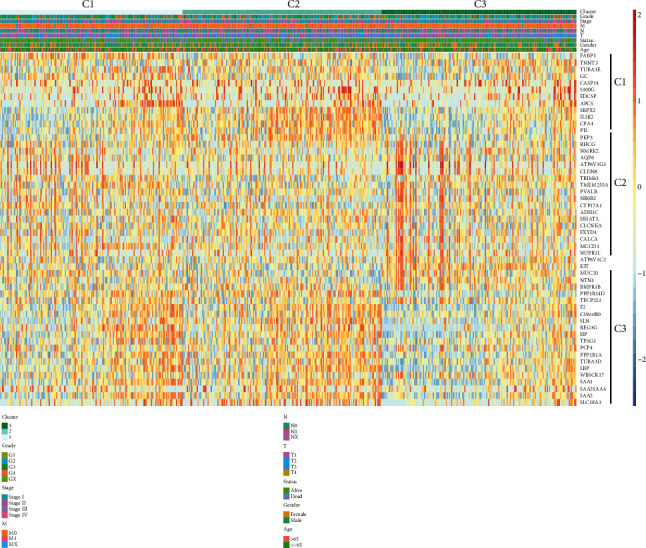
Correlation of clusters with clinicopathological characteristics. The heat map showing clinicopathological features of three clusters and gene expression of cluster-specific genes of each cluster. Expression data were normalized by log2 transformation.

We investigated the correlation between clinical characteristics and three clusters. The heatmap showed the expression levels of cluster-specific genes, and correlation between clusters and clinical characteristics including grade, AJCC stage, T stage, N stage, M stage, gender, age, and survival outcome (Figure 5). The results of the chi-square test revealed that C1, C2, and C3 were significantly associated with grade (*P* < 0.05) (Table 1). C1 and C2 correlated with advanced histologic grade while C3 was associated with histologic G1 and G2. But there was no significant difference between clusters and age, gender, T stage, N stage, M stage, and AJCC stage.

### 3.5. Correlation of the ccRCC Clusters with Immune Infiltration

**Figure 6:**
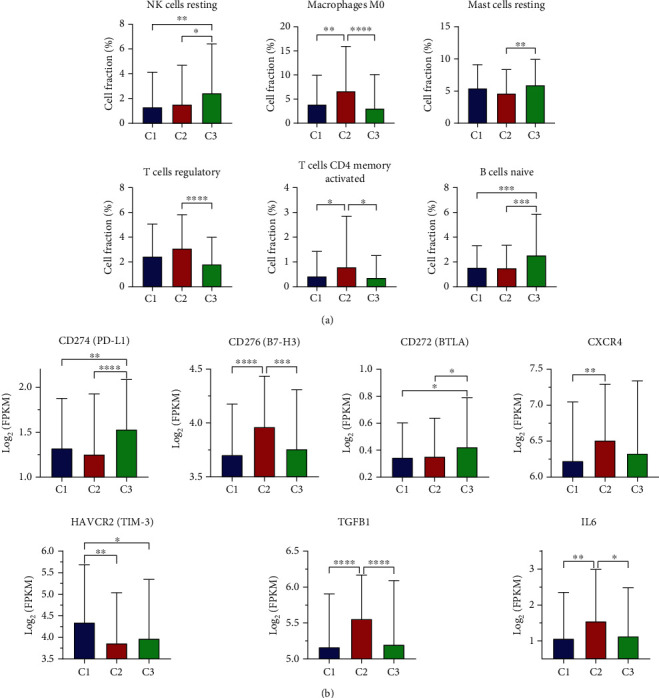
Correlation of the ccRCC clusters with immune infiltration estimated by the CIBERSORTx algorithm. (a) Differences in fraction of immune cells were compared among three clusters. (b) Expression levels of seven dysregulated immune checkpoint genes (CD274, CD276, CD272, CXCR4, HAVCR2, TGFB1, and IL-6) in three clusters. FPKM data were used and normalized by log2 transformation. ^∗^*P* < 0.05, ^∗∗^*P* < 0.01, ^∗∗∗^*P* < 0.001, and ^∗∗∗∗^*P* < 0.0001.

To characterize the differences of the immunologic landscape among clusters, the CIBERSORTx algorithm was used to estimate the immune cell infiltration of ccRCC samples. The results showed that there were significant differences in six immune cells including naive B cells, CD4^+^ memory activated T cells, regulatory T cells, resting NK cells, M0 macrophages, and resting mast cells among three clusters (Figure 6(a)). More resting NK cells and naive B cells enriched in C3 compared with C1 and C2. Additionally, C2 showed upregulated signature of M0 macrophages and activated memory CD4^+^ T cells and regulatory T cells compared with C1 and C3.

We then investigated immune checkpoint genes that played vital roles in immune regulation. Significant differences were found in CD274 (PD-L1), CD276 (B7-H3), CD272 (BTLA), CXCR4, HAVCR2 (TIM-3), TGFB1, and IL-6 (Figure 6(b)). The expression of PD-L1 and BTLA were significantly higher in C3, while novel immune checkpoint genes CD276, CXCR4, TGFB1, and IL-6 were upregulated in C2. Only HAVCR2 was higher in C1 compared with other clusters. However, the expression of other immune checkpoint genes such as PD-1, B7-H4, and CTLA4 did not differ among clusters. Collectively, we discovered significant differences in immune cell abundance and immune checkpoint genes among clusters.

### 3.6. Correlation of the ccRCC Clusters with Mutation, TMB, CYT, and Immune Response

**Figure 7:**
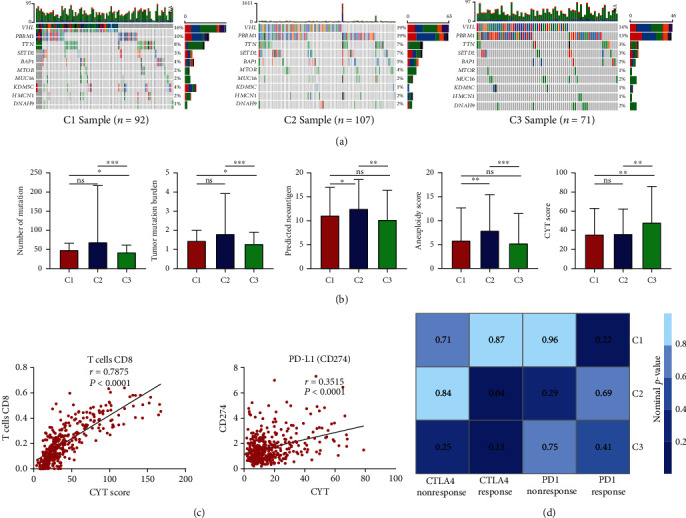
Association between three clusters and mutation, neoantigen, aneuploidy, cytolytic score (CYT), and immune response. (a) The oncoprint analyses of mutation status of top 10 genes in C1, C2, and C3. The proportion of mutated genes was shown on the right of each plot. (b) Comparison of the number of mutations, TMB, predicted neoantigen, aneuploidy score, and CYT score among three clusters. (c) The Spearman correlation analyses between the CYT score and CD8^+^ T cell abundance as well as PD-L1 expression. Correlation coefficient *r* and *P* values were shown. (d) Prediction of immune response of three clusters to anti-CTLA-4 and anti-PD-1 immunotherapy by the SubMap analysis. ^∗^*P* < 0.05, ^∗∗^*P* < 0.01, and ^∗∗∗^*P* < 0.001.

The link between metabolic alteration and gene mutation has been unraveled recently [27]. We investigated somatic mutation frequency among three clusters. Results showed that genes with high mutation frequency were similar in three clusters although different clusters tended to have different proportions for each mutated gene (Figure 7(a)). The top 10 mutated genes accounted for more proportion of overall mutation in C2 compared with C1 and C3. Moreover, it has been demonstrated that the overall mutation load and neoantigen load may drive T cell response [24, 28]. The number of mutation was calculated, and it showed that C2 had the most overall mutation numbers and TMB (Figure 7(b)). In terms of predicted neoantigen, C2 had more neoantigen loads than other clusters. Tumor aneuploidy is associated with reduced response to immunotherapy and inversely related to patient survival. Therefore, it might be used to help identify patients that possibly respond to immunotherapy [29]. The aneuploidy score was higher in C2 while C3 scored the lowest. In addition, the CYT estimated by average gene expression of GZMA and PRF1, represented the cytotoxic T cell activation [24]. Among various cancers, the ccRCC had high level of CYT, which were increased in response to CTLA-4 and PD-L1 immunotherapy as well as CD8^+^ T cell activation [30–32]. We analyzed the correlation of the CYT score with abundance of CD8^+^ T cells and the expression of PD-L1 (Figure 7(c)). The CYT score exhibited strong correlation with CD8^+^ T cells (Pearson's correlation *r* = 0.7875, *P* < 0.0001) and medium correlation with PD-L1 (Pearson's correlation *r* = 0.3515, *P* < 0.0001). Additionally, C3 had higher CYT score, indicating more cytotoxic T cell activation.

Considering the strong correlation between clusters and immune infiltration, the responses of ccRCC clusters to immune checkpoint anti-CTLA-4 and anti-PD-1 therapy were investigated using SubMap analysis, which could estimate the significance of association between subclasses. The expression profiles of our three clusters were used to compare with a published profile with immunotherapy response of 47 melanoma patients [25]. The SubMap analysis revealed that C2 showed significant correlation with the CTLA-4 response group, indicating that C2 patients might benefit from anti-CTLA-4 immunotherapy (Figure 7(d)). However, this prediction was inconsistent with aforementioned analyses, therefore more research was necessary to confirm the response of clusters to the immunotherapy.

### 3.7. Validation of DERG in the ccRCC

**Figure 8:**
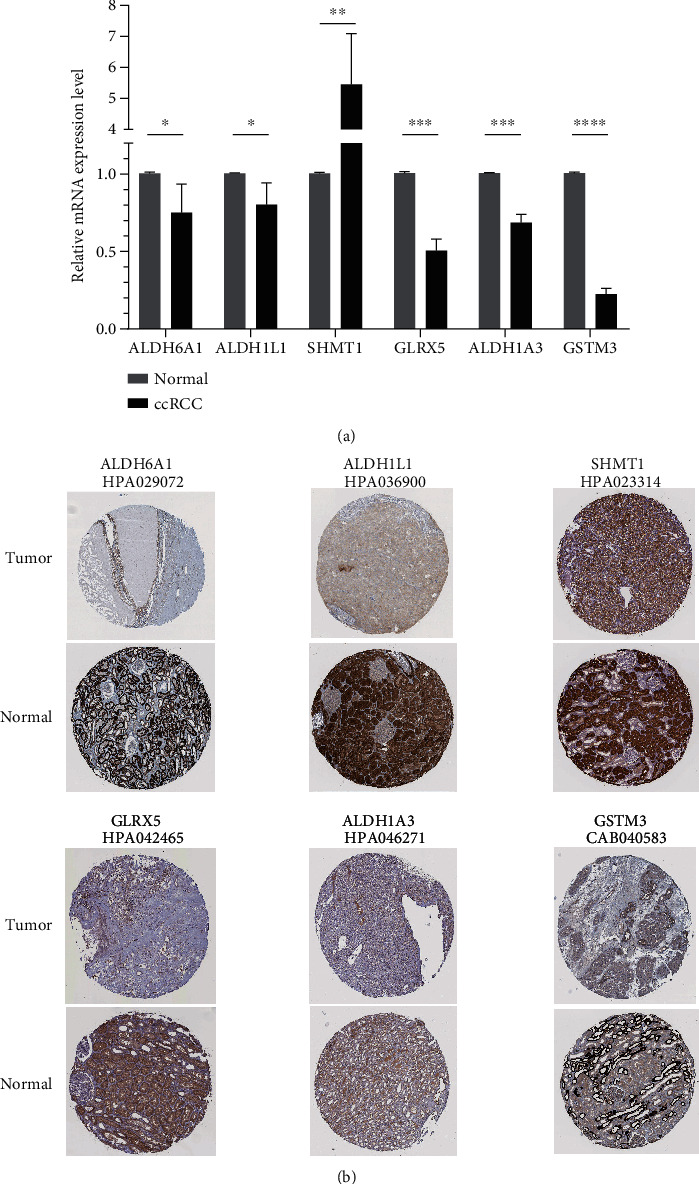
Validation of the mRNA and protein expression levels of six prognostic DERGs in ccRCC. (a) qRT-PCR results showed relative mRNA expression of ALDH6A1, ALDH1L1, SHMT1, GLRX5, ALDH1A3, and GSTM3 in ccRCC cell line (A498) and normal renal tubular epithelial cell (HK-2). GAPDH mRNA was used as an internal control. Data are presented as mean ± standard deviation. *n* = 3 in ccRCC and normal group. (b) The immunohistochemical results of ALDH6A1, ALDH1L1, SHMT1, GLRX5, ALDH1A3, and GSTM3 in ccRCC compared with normal kidney tissues from the HPA database. The antibody used was shown below the gene name. ^∗^*P* < 0.05, ^∗∗^*P* < 0.01, ^∗∗∗^*P* < 0.001, and ^∗∗∗∗^*P* < 0.0001.

To verify the expression level of the six prognostic DERGs, the mRNA expression level was further validated by qRT-PCR in normal renal tubular epithelial cell line (HK-2) and ccRCC cell line (A498). Consistent with the results of the database analysis, ALDH6A1, ALDH1L1, GLRX5, ALDH1A3, and GSTM3 were downregulated in ccRCC cell compared with normal cell (Figure 8(a)). But SHMT1 was upregulated in ccRCC. Moreover, the HPA database was used to analyze the protein expression of these prognostic DERGs. The immunohistochemical results showed that ALDH6A1, ALDH1L1, GLRX5, ALDH1A3, and GSTM3 were decreased in ccRCC compared with normal kidney tissues but there was no significant difference in SHMT1. Collectively, these results confirmed the low expression of prognostic DERGs except SHMT1 in ccRCC.

## 4. Discussion

Increasing evidence seeks to uncover molecular characteristics of cancer cells and propose molecular classification based on gene expression profiles [33–36]. However, no consensus has been reached in molecular classification of ccRCC. The present study identified DERGs and metabolism-regulating genes in ccRCC and investigated their prognostic value. Based on the 139 metabolic gene expression profile, ccRCC patients were stratified into three clusters (C1, C2, and C3). We explored the association of clusters with clinical traits, potential function, prognostic value, mutation, immune filtration, and immunotherapy efficacy. The GSVA results revealed that C1 had the most cluster-specific pathways which were mainly lipid metabolism, and thus, C1 was regarded as metabolic active subtype. Patients in C1 might respond to metabolic therapeutics and had moderate prognosis. Intermediate metabolic subclass C2 had higher aneuploidy score, TMB, neoantigen, and more infiltration of regulatory T cells. Besides, C2 patients tended to have advanced histologic grade and worse prognosis. But C2 was predicted to possibly respond to anti-CTLA-4 immunotherapy. Metabolic exhausted C3 exhibited higher CYT scores and better prognosis than C1 and C2. Collectively, this study presented a novel redox-associated classification of ccRCC, which could help uncover the heterogeneity of ccRCC and might be applied to improve therapeutic strategies.

Redox metabolism sustains normal cellular function and ensures cell survival, characterized by the production of ROS which exerts oxidative stress and renders dysfunctional cells to death [6, 37]. But it is recognized as the hallmark of cancer cells whose elevated antioxidant defense mechanisms are interfered by ROS, supporting the proliferation and survival of cancer cells [24]. In hepatocellular carcinoma, Benfeitas et al. highlighted substantial differences in redox metabolism and identified subtypes with various redox behavior based on redox genes [11]. Several studies had linked redox homeostasis to ccRCC. A nine redox-related lncRNA signature had been proposed to predict OS of ccRCC patients [38]. Additionally, renal cancer cells highly depended on the glutathione redox system to prevent lipid peroxidation and ferroptosis [10]. It has been reported that accumulation of fructose 1,6-bisphosphate and downregulation of aldolase B protected ccRCC from oxidative stress [10]. In our research, six prognostic DERGs including ALDH6A1, ALDH1L1, ALDH1A3, GSTM3, SHMT1, and GLRX5 were identified in ccRCC. Low expression of them was associated with worse prognosis while upregulated ALDH1A3 correlated with worse prognosis. The qRT-PCR results validated the low expression of these genes except SHMT1 which was increased in ccRCC cells. ALDH6A1 and ALDH1L1 which belong to the aldehyde dehydrogenase family were reported to correlate with poor prognosis and advanced stage of ccRCC [39, 40]. A polymorphism of GSTM3, GSTM3-rs1055259 could suppress ROS activity and prevent ccRCC progression [24]. However, no detailed research on SHMT1 and GLRX5 in ccRCC has been conducted.

Moreover, we obtained a list of vital genes in major metabolic pathways in cancer [13, 14] and investigated their expression levels and prognostic value in ccRCC. As drivers of the metabolic reprogramming in cancer, MYC, AKT1, and TP53 were overexpressed in ccRCC but KRAS was downregulated. Only AKT1 and KRAS were prognostic genes of ccRCC based on TCGA data. AKT1 was regarded as the major regulator in metabolism of cancer growth and KRAS could promote autophagy under metabolic stress [41]. Highly expressed G6PD in ccRCC could stimulate the growth and invasion of ccRCC through ROS-associated pathway [42]. BAP1, PBRM1, and SETD2 were characterized as regulators of metabolism which increased dependence on pentose phosphate shunt and reduced TCA cycle [14]. The downregulated expression of them were found in ccRCC tissues and closely correlated with worse prognosis. Furthermore, they were recurrently mutated in all three clusters of ccRCC. In the TCA cycle, alteration of FH determines lots of changes in cancer cellular metabolism such as glycolytic switch towards ROS [43]. We found that patients with downregulated FH had worse prognosis though more experiments on its function in ccRCC were needed. ACLY which converted citrate to cytosolic acetyl-CoA linked glucose metabolism to lipid synthesis and was highly expressed in ccRCC tissues. Diminished expression of ACLY was shown to correlate with poor prognosis but Hatzivassiliou et al. reported that ACLY inhibition could suppress the growth of advanced malignancies through glucose-dependent pathway [44]. With similar function to ACLY, ACACA decreased in ccRCC but no significant difference was found in the survival analysis. In ccRCC, VHL was the most frequently mutated gene and responsible for regulation of oxygen and iron sensing pathway that regulated HIF including HIF-1*α* and HIF-2*α* [45]. Alteration of VHL led to accumulation of HIF*α* subunits, whose activity influenced angiogenesis, metastasis, and invasion [46]. Additionally, we validated that downregulated FBP1 was associated with shorter OS in ccRCC. FBP1 was shown to interfere ccRCC progression through inhibiting the Warburg effect and inhibiting nuclear HIF function [47].

Consensus clustering analysis is an unsupervised clustering method applied to investigate subclasses [16]. It has been used to identify four stable subtypes with survival significance in both mRNA and miRNA expression profiles in ccRCC from TCGA [48]. Our research focused on redox-associated molecular classification and identified three distinct clusters with significant prognostic value and clinical association. Still, we found significant correlation of our clusters (C1, C2, and C3) with aforementioned TCGA mRNA clusters (*P* = 5.60*E* − 13) and miRNA clusters (*P* = 2.19*E* − 09; Supplementary Table S7). GSVA results revealed that metabolic active C1 with the most cluster-specific metabolic pathway. It was mainly involved in glucose, protein, and especially lipid metabolism including cholesterol metabolism, fat digestion, glycolipid, and cellular lipid catabolic processes. It has been reported that ccRCC showed metabolic reprogramming in terms of glucose, fatty acid metabolism, and tricarboxylic acid cycle [38]. The high enrichment in metabolic pathways indicated that C1 patients might benefit from metabolic therapies, providing alternatives for patients with unsatisfactory chemotherapy or immunotherapy. Moreover, C1 was associated with moderate histologic grade and moderate survival prognosis. These results suggested that C1 patients might have intermediate severity and prognosis and possibly respond to metabolic therapeutics. On the other hand, the intermediate metabolic subclass C2 was mainly involved in purine metabolism, pyrimidine metabolism, and glycan synthesis. Aneuploidy score and infiltration of regulatory T cells were inversely related to patient's survival in cancer [29]. Our results showed that C2 patients had the highest aneuploidy score and the most abundance of regulatory T cells. To some extent, this might contribute to the worst prognosis of C2 patients. Higher TMB followed by more neoantigen could increase T cell recognition and correlated with better response to immune checkpoint inhibitors [49]. C2 had more TMB, neoantigen, and high expression of several immune checkpoint genes (CD276, CXCR4, TGFB1, and IL-6), suggesting possible drug sensitivities towards immune checkpoint inhibitors [38]. Moreover, results of the SubMap analysis indicated that C2 might respond to anti-CTLA-4 therapy. The other cluster C3 had only two cluster-specific metabolic pathways, and thus, it was seen as metabolic exhausted subtype. It mainly correlated with lower histologic grade and the best prognosis. The removal of regulatory T cells could enhance antitumor immune response [50], and low infiltration of regulatory T cells found in C3 might suggest better response to immunotherapy. It was reported that CYT scores were positively correlated with prolonged survival in a variety of cancers [24], and higher CYT score in C3 suggested better prognosis than C1 and C2. Collectively, these results showed the heterogeneity of ccRCC and clarified differences in metabolism and immune in each cluster.

However, some limitations should be pointed out in this study. First, more datasets were necessary to validate robustness of our classification. Additional experiments should be carried out to validate potential function of DERGs and functional differences among clusters in ccRCC. It would be more convincing if clinical samples could be used for analysis, and large-scale clinical trials were needed to further validate the classification.

## 5. Conclusions

The present study stratified ccRCC patients into three clusters with distinct metabolic function and prognosis using redox gene expression profile. These three clusters exhibited significant differences in terms of immune infiltration, clinical traits, and mutation. Our classification might help predict prognosis of ccRCC patients and support the development of new therapeutic strategies.

## Figures and Tables

**Table 1:** Clinicopathological characteristics between three clusters in TCGA cohort.

Characteristics	Cluster 1 (*n*, %)	Cluster 2 (*n*, %)	Cluster 3 (*n*, %)	*P* value
Age				0.348
>65	62 (36.9%)	66 (36.1%)	54 (30.2%)	
≤65	106 (63.1%)	117 (63.9%)	125 (69.8%)	
Gender				0.574
Male	110 (65.5%)	123 (67.2%)	111 (62%)	
Female	58 (34.5%)	60 (32.8%)	68 (38%)	
T stage				0.116
T1	92 (54.6%)	84 (45.9%)	85 (53.1%)	
T2	17 (10.1%)	23 (12.6%)	29 (16.2%)	
T3	54 (33.5%)	70 (38.2)	53 (29.6%)	
T4	3 (1.8%)	6 (3.3%)	2 (1.1%)	
N stage				0.281
N0	83 (49.4%)	76 (41.5%)	80 (44.7%)	
N1	3 (1.8%)	9 (4.9%)	4 (2.2%)	
Nx	82 (48.8%)	98 (53.6%)	95 (53.1%)	
M stage				0.521
M0	140 (83.3%)	146 (79.8%)	154 (86%)	
M1	24 (14.3%)	34 (18.6%)	22 (12.3%)	
Mx	4 (2.4%)	3 (1.6%)	3 (1.7%)	
AJCC stage				0.059
Stage I	90 (53.6%)	81 (44.3%)	95 (53.1%)	
Stage II	13 (7.7%)	17 (9.3%)	27 (15.1%)	
Stage III	40 (23.8%)	49 (26.8%)	35 (19.6%)	
Stage IV	25 (14.9%)	36 (19.7%)	22 (12.3%)	
Grade				0.024
G1	4 (2.4%)	3 (1.6%)	7 (3.9%)	
G2	60 (35.7%)	76 (41.5%)	93 (52%)	
G3	78 (46.4%)	70 (38.3%)	59 (33%)	
G4	25 (14.9%)	33 (18%)	17 (9.5%)	
Gx	1 (0.6%)	1 (0.5%)	3 (0.9%)	

## Data Availability

The datasets analyzed for this study can be found in TCGA database (http://cancergenome.nih.gov/).

## Abbreviations

ccRCC: Clear cell renal cell carcinoma
RCC: Renal cell carcinoma
TCGA: The Cancer Genome Atlas
DEGs: Differentially expressed genes
DERGs: Differentially expressed redox genes
CTLA-4: Cytotoxic T lymphocyte associated protein-4
ROS: Reactive oxygen species
FPKM: Fragments per kilobase of transcript per million mapped reads
CDF: Cumulative distribution function
PCA: Principal component analysis
GO: Gene ontology
KEGG: Kyoto Encyclopedia of Genes and Genomes
TMB: Tumor mutation burden
CYT: Cytolytic activity
PD-1: Programmed cell death protein-1
qRT-PCR: Quantitative real-time PCR
OS: Overall survival
DFS: Disease-free survival
ALDH6A1: Aldehyde dehydrogenase 6 family member A1
ADLH1L1: Aldehyde dehydrogenase 1 family number L1
ALDH1A3: Aldehyde dehydrogenase 1 family number A3
SHMT1: Serine hydroxymethyltransferase 1
GLRX5: Glutaredoxin 5
GSTM3: Glutathione S-transferase mu 3
ACACA: Acetyl-CoA carboxylase alpha
FBP1: Fructose-bisphosphatase 1
BAP1: BRCA1 associated protein 1
FH: Fumarate hydratase
HIF1A: Hypoxia inducible factor 1 subunit alpha
HIF2A: Hypoxia inducible factor 2 subunit alpha
KRAS: KRAS proto-oncogene, GTPase
MTOR: Mechanistic target of rapamycin kinase
VHL: von Hippel-Lindau tumor suppressor
PBRM1: Polybromo 1
PRKAA1: Protein kinase AMP-activated catalytic subunit alpha 1
SETD2: SET domain containing 2 histone lysine methyltransferase
ACLY: ATP citrate lyase
AKT1: AKT serine/threonine kinase 1
MYC: MYC proto-oncogene
bHLH: Transcription factor
G6PD: Glucose-6-phosphate dehydrogenase
GSVA: Gene set variation analysis
HR: Hazard ratio.

## Supplementary Materials

Supplementary Materials Supplementary Table S1: primers used in the qRT-PCR for this study. Supplementary Table S2: 139 redox genes with median FPKM > 1. Supplementary Table S3: 31 differentially expressed redox genes. Supplementary Table S4: the list of vital genes for metabolism regulation. Supplementary Table S5: GSVA analysis in terms of metabolism-associated signatures. Supplementary Table S6: differential expression analysis and survival analysis of cluster-specific genes. Table S7: correlation between our clusters (C1, C2, and C3) and previously TCGA mRNA and miRNA clusters.
