# Supplementary material for: Redox Metabolism-Associated Molecular Classification of Clear Cell Renal Cell Carcinoma
Source: Oxid Med Cell Longev. 2022 Jan 21;2022:5831247. doi: 10.1155/2022/5831247 (PMC8799361; doi:10.1155/2022/5831247)
Supplement: Supplementary Materials — Supplementary Table S1: primers used in the qRT-PCR for this study. Supplementary Table S2: 139 redox genes with median FPKM > 1. Supplementary Table S3: 31 differentially expressed redox genes. Supplementary Table S4: the list of vital genes for metabolism regulation. Supplementary Table S5: GSVA analysis in terms of metabolism-associated signatures. Supplementary Table S6: differential expression analysis and survival analysis of cluster-specific genes. Table S7: correlation between our clusters (C1, C2, and C3) and previously TCGA mRNA and miRNA clusters. [file 5831247.f1.docx]

| Table S1: Primers used for this study | |  |
| --- | --- | --- |
| **Gene** | **Forward primer** | **Reverse primer** |
| ALDH6A1 | GGCAGACACTTCAGTATTAAGCC | AGAGGCAGACGGTAGGAATAAA |
| ALDH1L1 | TCAGTGCCAACTGTAAAGCTC | TGAGGGACCCGACCAATGA |
| SHMT1 | TGGCGGGACTGAGTTTATTGA | AGATGGACGTGGCAGAGATTT |
| GLRX5 | TCAGCAACGCCGTGGTGCAGA | TTGAGGTACACTTGCGGGATGG |
| ALDH1A3 | TGAATGGCACGAATCCAAGAG | CACGTCGGGCTTATCTCCT |
| GSTM3 | TACCTCTTATGAGGAGAAACGGT | AGGAAAGTCCAGGTCTAGCTTG |
| GAPDH | GGAGCGAGATCCCTCCAAAAT | GGCTGTTGTCATACTTCTCATGG |

| Table S2: 139 redox genes with median FPKM >1 | | |
| --- | --- | --- |
| **Gene** | **logFC** | **FDR** |
| ALDH16A1 | -0.01426 | 0.941509 |
| ALDH18A1 | 0.28856 | 5.53E-05 |
| ALDH1A1 | 0.139372 | 0.288149 |
| ALDH1A3 | -1.40011 | 5.16E-18 |
| ALDH1B1 | -1.27912 | 5.86E-26 |
| ALDH1L1 | -1.18271 | 5.87E-16 |
| ALDH2 | -0.85031 | 3.16E-24 |
| ALDH3A2 | -0.72721 | 5.48E-15 |
| ALDH3B1 | 0.704955 | 1.03E-09 |
| ALDH4A1 | -2.08341 | 6.54E-52 |
| ALDH5A1 | -1.06725 | 4.59E-33 |
| ALDH6A1 | -3.04048 | 1.1E-184 |
| ALDH7A1 | -0.73297 | 3.88E-30 |
| ALDH8A1 | -0.94952 | 2.56E-07 |
| ALDH9A1 | -0.77219 | 2.21E-40 |
| CAT | -1.28909 | 4.32E-52 |
| CYBB | 1.788088 | 1.58E-24 |
| DHFR | -0.23082 | 0.028324 |
| FASN | 0.13182 | 0.250603 |
| FOXO1 | -0.7012 | 1.52E-14 |
| FOXO3 | 0.063544 | 0.486107 |
| FOXO4 | -0.81142 | 1.38E-39 |
| FPGS | 0.045372 | 0.464886 |
| G6PD | 0.218992 | 0.018934 |
| GCLC | 0.113809 | 0.284846 |
| GCLM | 0.224963 | 0.021107 |
| GGCT | -0.34727 | 5.86E-07 |
| GGT1 | 0.566239 | 0.000262 |
| GGT5 | -0.45242 | 0.00072 |
| GGT7 | -0.23612 | 0.002419 |
| GLRX | 0.256329 | 0.018984 |
| GLRX2 | 0.095974 | 0.299908 |
| GLRX3 | 0.130603 | 0.067697 |
| GLRX5 | -1.12276 | 1.96E-50 |
| GLUD1 | -0.5213 | 1.88E-13 |
| GLUD2 | -0.4312 | 0.000221 |
| GPX1 | 0.843959 | 3.72E-13 |
| GPX3 | -1.62627 | 7.17E-31 |
| GPX4 | 0.519527 | 9.42E-07 |
| GPX7 | 0.519675 | 7.06E-07 |
| GPX8 | 0.824546 | 2.22E-12 |
| GSR | -0.59841 | 1.89E-15 |
| GSS | -0.04225 | 0.616913 |
| GSTA1 | -0.02089 | 0.913143 |
| GSTA2 | -0.48198 | 0.042426 |
| GSTA4 | 0.019247 | 0.862983 |
| GSTK1 | 0.149761 | 0.066373 |
| GSTM1 | 0.593276 | 0.054009 |
| GSTM2 | 0.019456 | 0.941509 |
| GSTM3 | -3.30954 | 2.4E-268 |
| GSTM4 | -0.37026 | 8.96E-07 |
| GSTO1 | 0.433218 | 2.24E-05 |
| H6PD | 0.720555 | 2.27E-19 |
| HIF1A | -1.18936 | 4.52E-46 |
| HMOX1 | 2.319544 | 1.38E-48 |
| IDH1 | -0.66087 | 6.08E-14 |
| IDH2 | -1.11215 | 1.54E-38 |
| IDH3A | -0.54707 | 9.54E-08 |
| IDH3B | -0.00611 | 0.933452 |
| IDH3G | -0.21858 | 0.000329 |
| MDH1 | -0.85269 | 9.77E-38 |
| MDH2 | -0.40099 | 1.41E-08 |
| ME1 | 0.164214 | 0.300362 |
| ME2 | 0.127873 | 0.170079 |
| ME3 | 0.073282 | 0.480067 |
| MTHFD1 | -0.39214 | 2.59E-10 |
| MTHFD1L | 0.575876 | 4.22E-09 |
| MTHFD2 | 0.962658 | 1.18E-13 |
| NFE2L2 | -0.50615 | 4.48E-22 |
| NNT | -1.25603 | 6.1E-30 |
| NOX4 | -1.30451 | 5.96E-22 |
| NQO1 | -0.42662 | 0.000178 |
| PARK7 | -0.10129 | 0.111125 |
| PGD | -0.83663 | 1.07E-34 |
| PRDX1 | -0.54252 | 1.45E-21 |
| PRDX2 | -0.45329 | 1.14E-06 |
| PRDX3 | -0.90907 | 9.17E-45 |
| PRDX4 | 1.39899 | 1.42E-54 |
| PRDX5 | -0.1153 | 0.250745 |
| PRDX6 | -0.38543 | 2.42E-11 |
| RRM1 | 0.072083 | 0.257738 |
| SESN1 | 0.193824 | 0.03245 |
| SESN2 | -0.5805 | 1.41E-13 |
| SESN3 | -0.55021 | 1.75E-06 |
| SOD1 | -0.46168 | 9.18E-13 |
| SOD2 | 1.19601 | 4.57E-18 |
| SOD3 | -1.54846 | 3.72E-46 |
| SQSTM1 | 0.547184 | 4.22E-12 |
| TP53 | 0.459449 | 4.2E-15 |
| TXN | 0.203684 | 0.045316 |
| TXN2 | -0.40402 | 1.37E-09 |
| TXNDC11 | 0.069354 | 0.232109 |
| TXNDC12 | 0.384099 | 1.67E-13 |
| TXNDC15 | 0.347682 | 2.24E-10 |
| TXNDC16 | -0.08711 | 0.346938 |
| TXNDC17 | -0.02214 | 0.899765 |
| TXNDC5 | 0.490961 | 0.000964 |
| TXNDC9 | -0.26866 | 0.000113 |
| TXNL1 | -0.085 | 0.329176 |
| TXNL4A | 0.061563 | 0.346615 |
| TXNL4B | 0.106075 | 0.297426 |
| TXNRD1 | 0.001886 | 0.998847 |
| TXNRD2 | -0.55848 | 6.8E-09 |
| TXNRD3 | -0.41837 | 0.000141 |
| ACSS1 | -0.81108 | 8.66E-19 |
| ACSS2 | -0.73345 | 1.76E-23 |
| ALDOA | 1.082519 | 4.38E-42 |
| ALDOB | -4.74703 | 1.05E-85 |
| ALDOC | 2.240468 | 1.48E-38 |
| AMT | -1.07257 | 8.18E-42 |
| ATIC | -0.08186 | 0.33011 |
| CD44 | 1.377631 | 1.81E-16 |
| FBP1 | -2.35348 | 2.95E-78 |
| GAPDH | 1.450902 | 2.47E-52 |
| GART | -0.18959 | 0.014493 |
| GGH | -1.42009 | 2.72E-36 |
| GLDC | -2.32394 | 3.89E-74 |
| GLS | -0.99178 | 4.33E-38 |
| GPI | 0.513081 | 1.57E-12 |
| MTHFR | 0.203114 | 0.054672 |
| MTHFS | -0.13029 | 0.141562 |
| MTR | -0.22028 | 0.000333 |
| OPLAH | -0.5456 | 5.17E-11 |
| PCYOX1 | -0.86683 | 6.68E-42 |
| PFKL | 0.255578 | 0.000182 |
| PFKM | -0.99646 | 5.19E-27 |
| PFKP | 1.947286 | 5.99E-72 |
| PGLS | 0.814514 | 4.29E-19 |
| PGM1 | 0.276573 | 0.000558 |
| PGM2 | 0.002208 | 1 |
| PKLR | -0.87663 | 9.91E-06 |
| PKM | 0.564967 | 2.41E-14 |
| PRPS1 | 0.005301 | 0.997193 |
| PRPS2 | -0.98888 | 1.05E-34 |
| RPE | -0.30178 | 2.52E-07 |
| SHMT1 | -1.04972 | 5.67E-33 |
| SHMT2 | 1.928053 | 2.72E-78 |
| TALDO1 | -0.14287 | 0.08076 |
| TKT | 0.146584 | 0.122075 |

| TableS3: 31 differentially expressed redox genes | | |
| --- | --- | --- |
| **gene** | **logFC** | **FDR** |
| ALDH1A3 | -1.40011 | 5.16E-18 |
| ALDH1B1 | -1.27912 | 5.86E-26 |
| ALDH1L1 | -1.18271 | 5.87E-16 |
| ALDH4A1 | -2.08341 | 6.54E-52 |
| ALDH5A1 | -1.06725 | 4.59E-33 |
| ALDH6A1 | -3.04048 | 1.14E-184 |
| CAT | -1.28909 | 4.32E-52 |
| CYBB | 1.788088 | 1.58E-24 |
| GLRX5 | -1.12276 | 1.96E-50 |
| GPX3 | -1.62627 | 7.17E-31 |
| GSTM3 | -3.30954 | 2.35E-268 |
| HIF1A | -1.18936 | 4.52E-46 |
| HMOX1 | 2.319544 | 1.38E-48 |
| IDH2 | -1.11215 | 1.54E-38 |
| NNT | -1.25603 | 6.1E-30 |
| NOX4 | -1.30451 | 5.96E-22 |
| PRDX4 | 1.39899 | 1.42E-54 |
| SOD2 | 1.19601 | 4.57E-18 |
| SOD3 | -1.54846 | 3.72E-46 |
| ALDOA | 1.082519 | 4.38E-42 |
| ALDOB | -4.74703 | 1.05E-85 |
| ALDOC | 2.240468 | 1.48E-38 |
| AMT | -1.07257 | 8.18E-42 |
| CD44 | 1.377631 | 1.81E-16 |
| FBP1 | -2.35348 | 2.95E-78 |
| GAPDH | 1.450902 | 2.47E-52 |
| GGH | -1.42009 | 2.72E-36 |
| GLDC | -2.32394 | 3.89E-74 |
| PFKP | 1.947286 | 5.99E-72 |
| SHMT1 | -1.04972 | 5.67E-33 |
| SHMT2 | 1.928053 | 2.72E-78 |

| Table S4: vital genes for metabolism regulation from Massari et al and Tong et al | |
| --- | --- |
| **Gene symbol** | **Function** |
| VHL | Angiogenesis, ECM remodeling, genomic integrity, glycolytic enzyme expression, anabolic biosynthesis, cell cycle progression |
| HIF1A |  |
| HIF2A |  |
| GLUT1(SLC2A1) |  |
| MYC |  |
| G6PD | Pentose phosphate pathway, fatty acids and cholesterol biosynthesis |
| ACLY | Glucose metabolism to lipid synthesis |
| ACACA |  |
| BAP1 | Pentose phosphate shunt, lipogenesis, AMPK and Krebs cycle activity |
| PBRM1 |  |
| SETD2 |  |
| FBP1 | Metabolism |
| FH | Energy production, angiogenesis, cell growth, cell proliferation, oxidative stress |
| MTOR | Survival, growth, proliferation, motility, metabolism, energy balance, stress response and angiogenesis, adipogenesis |
| AKT (AKT1) | directly enhances aerobic glycolysis; facilitate the coupling of glycolysis and OXPHOS |
| AMPK (PRKAA1) | Fatty acid synthesis, mTOR pathway |
| KRAS | activation can promote scavenging pathways such as autophagy and macropinocytosis in cancer cells under metabolic stress |
| TP53 | loss of TP53 was reminiscent of the “Warburg effect” (aerobic glycolysis) |

| Table S5: GSVA analysis in terms of metabolism associated signatures | | | | |
| --- | --- | --- | --- | --- |
| **Terms** | C1 VS C2 | C1 VS C3 | C2 VS C3 | adj.P.Val |
| Vitamin K | -0.27265 | 0.549329 | 0.821983 | 3.87E-39 |
| Galactose Metabolism | 0.027046 | 0.276761 | 0.249715 | 3.69E-30 |
| Purine Metabolism | -0.04628 | 0.097381 | 0.14366 | 3.84E-30 |
| Glutathione Metabolism | 0.06477 | 0.260075 | 0.195305 | 3.84E-30 |
| Pentose Phosphate | 0.022431 | 0.255412 | 0.23298 | 3.84E-30 |
| Fructose and Mannose Metabolism | 0.062065 | 0.316405 | 0.25434 | 1.59E-29 |
| Amino Sugar and Nucleotide Sugar Metabolism | 0.042207 | 0.275915 | 0.233708 | 5.55E-27 |
| Pyrimidine Metabolism | -0.06728 | 0.132946 | 0.200224 | 3.34E-25 |
| Pyrimidine Biosynthesis | -0.11019 | 0.220166 | 0.330353 | 7.17E-23 |
| Drug Metabolism by other enzymes | 0.02143 | 0.182372 | 0.160943 | 3.03E-22 |
| Porphyrin and Chlorophyll Metabolism | 0.052787 | 0.239533 | 0.186746 | 9.99E-22 |
| Riboflavin Metabolism | -0.0896 | 0.425195 | 0.514795 | 1.3E-21 |
| Arachidonic Acid Metabolism | 0.06751 | 0.178114 | 0.110604 | 6.86E-21 |
| Glycine, Serine and Threonine Metabolism | 0.16109 | 0.289208 | 0.128118 | 8.38E-20 |
| Sulfur Metabolism | 0.025668 | 0.323221 | 0.297554 | 1.83E-19 |
| Ubiquinone and other Terpenoid-Quinone Biosynthesis | -0.10661 | 0.21542 | 0.322027 | 1.83E-19 |
| Gluconeogenesis | 0.111976 | 0.260516 | 0.148539 | 5.24E-19 |
| Metabolism of Xenobiotics by Cytochrome P450 | 0.108843 | 0.201795 | 0.092952 | 9.6E-19 |
| Tyrosine Metabolism | 0.085118 | 0.210832 | 0.125714 | 1.69E-18 |
| Glycolysis | 0.089475 | 0.246439 | 0.156964 | 2.69E-18 |
| Phenylalanine Metabolism | 0.122904 | 0.254401 | 0.131497 | 5.89E-17 |
| Drug Metabolism by Cytochrome P450 | 0.119399 | 0.190822 | 0.071423 | 1.02E-16 |
| Starch and Suctose Metabolism | -0.00313 | 0.167573 | 0.170699 | 1.32E-16 |
| Folate One Carbon Metabolism | 0.088692 | 0.218793 | 0.130101 | 2.31E-16 |
| Alanine, Aspartate and Glutamate Metabolism | 0.105415 | 0.217585 | 0.11217 | 9.35E-16 |
| Inositol Phosphate Metabolism | 0.074612 | -0.08866 | -0.16327 | 2.15E-15 |
| Primary Bile Acid Biosynthesis | 0.103457 | 0.254688 | 0.151231 | 2.59E-15 |
| Tryptophan Metabolism | 0.213036 | 0.228109 | 0.015073 | 8.94E-15 |
| Glyoxylate and Dicarboxylate Metabolism | 0.225388 | 0.271997 | 0.046609 | 1.27E-14 |
| Pentose and Glucuronate Interconversions | 0.142448 | 0.229547 | 0.087099 | 2.12E-14 |
| Arginine and Proline Metabolism | 0.092561 | 0.179852 | 0.087291 | 4.5E-14 |
| Oxidative Phosphorylation | 0.147363 | 0.32232 | 0.174958 | 5.89E-14 |
| Heme Biosynthesis | 0.015916 | 0.272244 | 0.256327 | 1.26E-13 |
| Fatty Acid Elongation | 0.137971 | 0.220431 | 0.08246 | 1.35E-13 |
| Pantothenate and CoA Biosynthesis | 0.098173 | 0.18352 | 0.085347 | 1.35E-13 |
| Valine, Leucine and Isoleucine Biosynthesis | 0.003065 | 0.314676 | 0.311611 | 2.07E-13 |
| Biosynthesis of Unsaturated Fatty Acids | 0.140925 | 0.229953 | 0.089028 | 2.07E-13 |
| Beta-Alanine Metabolism | 0.17772 | 0.215885 | 0.038165 | 2.79E-13 |
| Dopamine Biosynthesis | 0.180698 | 0.320432 | 0.139733 | 4.81E-13 |
| Steroid Hormone Metabolism | 0.047078 | 0.144616 | 0.097539 | 6.77E-13 |
| Fatty Acid Degradation | 0.245615 | 0.208038 | -0.03758 | 6.77E-13 |
| Other Types of O-Glycan Biosynthesis | -0.13839 | 0.048032 | 0.186424 | 8.32E-13 |
| Butanoate Metabolism | 0.238713 | 0.218124 | -0.02059 | 1.05E-12 |
| Prostaglandin Biosynthesis | 0.026582 | 0.220169 | 0.193588 | 1.4E-12 |
| Folate biosynthesis | 0.119199 | 0.258732 | 0.139533 | 2.2E-12 |
| Testosterone Biosynthesis | 0.05551 | 0.207628 | 0.152118 | 2.79E-12 |
| Histidine Metabolism | 0.235627 | 0.202129 | -0.0335 | 2.98E-12 |
| Norepinephrine Biosynthesis | 0.244271 | 0.314205 | 0.069933 | 3.5E-12 |
| Aldosterone Biosynthesis | 0.036178 | 0.245782 | 0.209604 | 5.09E-12 |
| Epinephrine Biosynthesis | 0.233891 | 0.255346 | 0.021455 | 5.99E-12 |
| Pyruvate Metabolism | 0.180364 | 0.202626 | 0.022262 | 1.23E-11 |
| Valine, Leucine and Isoleucine Degradation | 0.269695 | 0.222727 | -0.04697 | 1.35E-11 |
| Kynurenine Metabolism | 0.094831 | 0.238987 | 0.144156 | 1.45E-11 |
| Arginine Biosynthesis | 0.145117 | 0.192138 | 0.047022 | 1.6E-11 |
| Glycosphingolipid Biosynthesis | -0.01465 | 0.119117 | 0.13377 | 2.12E-11 |
| Cortisol Biosynthesis | 0.086062 | 0.222557 | 0.136495 | 4.2E-11 |
| Steroid Hormone Biosynthesis | 0.056682 | 0.13383 | 0.077148 | 4.91E-11 |
| Citric Acid Cycle | 0.248426 | 0.239345 | -0.00908 | 7.63E-11 |
| Nicotinamide Adenine Dinucleotide Biosynthesis | 0.130534 | 0.226763 | 0.096229 | 1.24E-10 |
| Retinol Metabolism | 0.037174 | 0.129242 | 0.092068 | 1.57E-10 |
| Methionine Cycle | -0.01305 | 0.085887 | 0.098937 | 5.09E-10 |
| Ketone Biosynthesis and Metabolism | 0.205303 | 0.18259 | -0.02271 | 7.79E-10 |
| Cysteine and Methionine Metabolism | 0.08074 | 0.154853 | 0.074113 | 1.05E-09 |
| Vitamin B6 Metabolism | 0.175587 | 0.25533 | 0.079743 | 1.41E-09 |
| Ascorbate and Aldrate Metabolism | 0.177486 | 0.184818 | 0.007332 | 1.77E-09 |
| Propanoate Metabolism | 0.252564 | 0.178461 | -0.0741 | 1.98E-09 |
| Phenylalanine, Tyrosine and Tryptophan Biosynthesis | 0.086273 | 0.266926 | 0.180653 | 2.7E-09 |
| Nicotinate and Nicotinamide Metabolism | 0.070387 | 0.129858 | 0.059471 | 3.14E-09 |
| Neomycin, Kanamysin and Gentamicin Biosynthesis | -0.09062 | 0.205973 | 0.296595 | 3.76E-09 |
| Glycosaminoglycan Degradation | -0.02522 | 0.144514 | 0.169735 | 3.79E-09 |
| Steroid Biosynthesis | 0.043451 | 0.163389 | 0.119939 | 7.98E-09 |
| Glycosphosphatidylinositol | 0.022592 | 0.166712 | 0.14412 | 9.45E-09 |
| Remethylation | 0.212623 | 0.204081 | -0.00854 | 8.08E-08 |
| Caffiene Metabolism | 0.149816 | 0.234274 | 0.084458 | 1.02E-07 |
| Glycogen Biosynthesis | -0.07864 | 0.067375 | 0.146017 | 1.03E-07 |
| Homocysteine Biosynthesis | -0.07345 | 0.059945 | 0.133395 | 2.06E-07 |
| Estradiol Biosynthesis | 0.051725 | 0.141231 | 0.089506 | 2.43E-07 |
| Retinoid Metabolism | -0.10736 | 0.022401 | 0.129761 | 9.36E-07 |
| N-Glycan Biosynthesis | -0.01881 | 0.100466 | 0.119274 | 1.44E-06 |
| Sirtuin Nicotinamide Metabolism | 0.016844 | 0.236169 | 0.219325 | 1.67E-06 |
| Glycosaminoglycan Biosynthesis | -0.12937 | -0.01252 | 0.116851 | 3.19E-06 |
| alpha-Linoleic Acid Metabolism | 0.052081 | 0.122209 | 0.070127 | 3.85E-06 |
| Purine Biosynthesis | -0.02238 | 0.105668 | 0.128049 | 8.22E-06 |
| Thiamine Metabolism | 0.039511 | 0.218609 | 0.179098 | 0.000011 |
| Shingolipid Metabolism | 0.090639 | 0.100286 | 0.009647 | 1.17E-05 |
| Nicotinamide Adenine Metabolism | 0.14777 | 0.154994 | 0.007224 | 1.33E-05 |
| Glycerolipid Metabolism | 0.075834 | 0.062612 | -0.01322 | 1.94E-05 |
| Urea Cycle | 0.069667 | 0.128225 | 0.058559 | 2.89E-05 |
| Lysine Degradation | 0.091007 | 0.009603 | -0.0814 | 7.76E-05 |
| Other Glycan Degradation | 0.08766 | 0.139985 | 0.052325 | 0.000125 |
| Cyclooxygenase Arachidonic Acid Metabolism | -0.16986 | -0.06489 | 0.104966 | 0.000178 |
| Selenocompound Metabolism | 0.0738 | 0.117885 | 0.044084 | 0.000182 |
| Fatty Acid Biosynthesis | 0.10349 | 0.030102 | -0.07339 | 0.00025 |
| Retinoic Acid Metabolism | -0.01438 | 0.087544 | 0.101926 | 0.000436 |
| Cholesterol Biosynthesis | 0.047216 | 0.110148 | 0.062932 | 0.000695 |
| Biotin Metabolism | 0.10511 | 0.204794 | 0.099683 | 0.002957 |
| Polyamine Biosynthesis | -0.0554 | 0.020354 | 0.075751 | 0.003218 |
| Lipoic Acid Metabolism | 0.014533 | 0.171502 | 0.156969 | 0.005587 |
| Linoleic Acid Metabolism | 0.045185 | 0.069463 | 0.024278 | 0.006179 |
| Cardiolipin Metabolism | -0.1176 | 0.009857 | 0.127454 | 0.008881 |
| Taurine and Hypotaurine Metabolism | -0.06968 | -0.01262 | 0.057069 | 0.010888 |
| Glycerophospholipid Metabolism | 0.03845 | 0.046016 | 0.007566 | 0.011575 |
| Transsulfuration | -0.07051 | -0.11279 | -0.04228 | 0.025965 |
| D-Glutamine and D-Glutamate Metabolism | 0.167418 | 0.096359 | -0.07106 | 0.027935 |
| Cardiolipin Biosynthesis | -0.02293 | 0.098045 | 0.120971 | 0.043155 |
| Ether Lipid Metabolism | 0.022678 | 0.047915 | 0.025237 | 0.043526 |

| Table S6: Differential expression analysis and survival analysis of cluster-specific genes | | | |
| --- | --- | --- | --- |
| Gene | logFC | FDR | P value of Survival analysis |
| **C1** |  |  |  |
| FABP1 | -4.378 | 0.000 | 0.001 |
| TNNT3 | 2.637 | 0.000 | 0.120 |
| TUBA3E | 1.893 | 0.000 | 0.067 |
| GC | 0.769 | 0.141 | 0.830 |
| CASP14 | 4.429 | 0.000 | 0.491 |
| S100G | 3.888 | 0.000 | 0.050 |
| FDCSP | 5.558 | 0.000 | 0.000 |
| APCS | -0.397 | 0.431 | 0.820 |
|  |  |  |  |
| **C2** |  |  |  |
| SRPX2 | -0.117 | 0.660 | 0.000 |
| IL1R2 | 1.097 | 0.001 | 6e−04 |
| RHCG | -1.058 | 0.049 | 0.240 |
| NMRK2 | 4.359 | 0.000 | 0.790 |
| AQP6 | -0.887 | 0.113 | 0.890 |
| CPA4 | 2.336 | 0.000 | 0.000 |
| PI3 | 0.145 | 0.785 | 4.2e−07 |
| ATP6V1G3 | -1.208 | 0.028 | 0.182 |
| PKP3 | -0.174 | 0.630 | 9.1e−06 |
| CLDN8 | -3.140 | 0.000 | 0.790 |
| TRIM63 | 2.441 | 0.000 | 0.150 |
| TMEM255A | 1.994 | 0.000 | 0.290 |
| PVALB | 0.271 | 0.736 | 0.150 |
| NR0B2 | -2.740 | 0.000 | 0.580 |
| CYP17A1 | -1.919 | 0.000 | 0.170 |
| ADH1C | -3.016 | 0.000 | 0.930 |
| HHATL | 4.790 | 0.000 | 0.032 |
| CLCNKA | -4.972 | 0.000 | 0.000 |
| FXYD4 | -6.210 | 0.000 | 0.820 |
| CALCA | -2.120 | 0.000 | 0.364 |
| MCCD1 | -4.194 | 0.000 | 0.013 |
| NUPR1L | -1.974 | 0.000 | 0.250 |
|  |  |  |  |
| **C3** |  |  |  |
| ATP6V1C2 | -1.870 | 0.000 | 1.2e−09 |
| KIT | -0.687 | 0.002 | 0.000 |
| MUC20 | -0.341 | 0.181 | 0.000 |
| NTN1 | -0.311 | 0.292 | 0.022 |
| BMPR1B | -1.972 | 0.000 | 0.340 |
| PPP1R14D | 2.026 | 0.000 | 0.550 |
| TFCP2L1 | -3.083 | 0.000 | 0.150 |
| F2 | 3.113 | 0.000 | 2.2e−06 |
| C19orf80 | 3.380 | 0.000 | 0.000 |
| SLN | 2.213 | 0.000 | 1.1e−06 |
| REG3G | 1.274 | 0.014 | 0.800 |
| HP | 6.677 | 0.000 | 0.002 |
| TPSG1 | 2.309 | 0.000 | 0.007 |
| PCP4 | -2.370 | 0.000 | 0.370 |
| PPP1R1A | -2.687 | 0.000 | 3.3e−06 |
| TUBA3D | 2.913 | 0.000 | 0.510 |
| LBP | 4.503 | 0.000 | 0.001 |
| WBSCR17 | -0.167 | 0.679 | 0.650 |
| SAA1 | 4.943 | 0.000 | 4.3e−09 |
| SAA2-SAA4 | 4.393 | 0.000 | 3.8e−08 |
| SAA2 | 4.506 | 0.000 | 4.4e−10 |
| SLC18A3 | 3.634 | 0.000 | 0.005 |
| CRP | 4.778 | 0.000 | 0.000 |

Table S7: Correlation between our clusters (C1, C2, C3) and previously TCGA mRNA and miRNA clusters.

| TCGA clusters | Redox-associated clusters | | | P value |
| --- | --- | --- | --- | --- |
|  | C1 | C2 | C3 |  |
| mRNA clusters |  |  |  | 5.60E-13 |
| m1 | 51(37.5%) | 29(20.4%) | 67(48.9%) |  |
| m2 | 26(19.1%) | 20(14.1%) | 43(31.4%) |  |
| m3 | 22(16.2%) | 51(35.9%) | 21(15.3%) |  |
| m4 | 37(27.2%) | 42(29.6%) | 6(4.4%) |  |
| miRNA clusters |  |  |  | 2.19E-09 |
| mi1 | 30(21.7%) | 10(7%) | 41(31.8%) |  |
| mi2 | 32(23.2%) | 65(45.5%) | 16(12.4%) |  |
| mi3 | 53(38.4%) | 43(30%) | 47(36.4%) |  |
| mi4 | 23(16.7%) | 25(17.5%) | 25(19.4%) |  |
| Chi-Square tests were performed to analyze the differences between redox-associated clusters (C1, C2, C3) and TCGA mRNA clusters (m1, m2, m3, m4) and miRNA clusters (mi1, mi2, mi3, mi4). The P value of Pearson Chi-Square was shown. Red = significant overlap. | | | | |
